# Immunosilencing peptides by stereochemical inversion and sequence reversal: *retro*-D-peptides

**DOI:** 10.1038/s41598-018-24517-6

**Published:** 2018-04-24

**Authors:** Pol Arranz-Gibert, Sonia Ciudad, Jesús Seco, Jesús García, Ernest Giralt, Meritxell Teixidó

**Affiliations:** 1 Institute for Research in Biomedicine (IRB Barcelona), Barcelona Institute of Science and Technology (BIST), Baldiri Reixac 10, Barcelona, E-08028 Spain; 2 Department of Inorganic and Organic Chemistry, University of Barcelona, Martí i Franquès 1-11, Barcelona, E-08028 Spain

## Abstract

Peptides are experiencing a new era in medical research, finding applications ranging from therapeutics to vaccines. In spite of the promising properties of peptide pharmaceuticals, their development continues to be hindered by three weaknesses intrinsic to their structure, namely protease sensitivity, clearance through the kidneys, and immune system activation. Here we report on two *retro*-D-peptides (H_2_N-hrpyiah-CONH_2_ and H_2_N-pwvpswmpprht-CONH_2_), which are protease-resistant and retain the original BBB shuttle activity of the parent peptide but are much less immunogenic than the parent peptide. Hence, we envisage that *retro*-D-peptides, which display a similar topological arrangement as their parent peptides, will expand drug design and help to overcome factors that lead to the failure of peptide pharmaceuticals in pre- and clinical trials. Furthermore, we reveal requirements to avoid or elicit specific humoral responses to therapeutic peptides, which might have a strong impact in both vaccine design and peptide therapeutic agents.

## Introduction

During the last three decades, peptides have become privileged therapeutics^1^ and are now used in a broad range of applications. The relevant presence of these molecules in nature and accessible synthesis through well-established solid-phase peptide synthesis (SPPS)^2^ has facilitated their study and applicability in the pharma industry^3^. Compared to therapeutics developed by classical medicinal chemistry (low molecular weight organic molecules), peptides display a better solubility profile, although this depends on the sequence^4^. While the abundance of peptides in nature is advantageous from the perspective of toxicity, two principal drawbacks are intrinsic to their structure, namely protease degradation driven by proteolytic enzymes and eventual risk of immunogenicity^1^. In addition, this class of therapeutics displays a short plasma half-life as a result of kidney clearance. Nevertheless, several approaches have partially resolved these issues. Recognition by proteases, and thus peptide degradation, can be avoided by using peptide derivatives like D-, β-^5^ or non-natural amino acid peptides, as well as other classes of peptidomimetics^6^. Furthermore, PEGylation^7^ and other methodologies^8^ can extend the plasma half-life of peptides by increasing their molecular weight (more precisely, their hydrodynamic radius).

The immunogenicity of peptides (as the native ligands of MHC class I/II) have been studied extensively^9^. While vaccine development requires activation of the immune response^10^, other therapeutic treatments require not to elicit such type of response. Few approaches have reported effective reduction of immune system recognition/response. PEGylation^7^ is probably the most widely used method of choice to reduce immunogenicity, although adverse immunological effects, *i*.*e*. humoral responses, have been reported^11^.

*Retro*-D-peptides (containing *retro*-inverso- and *retro*-enantio-isomers of a parent peptide)^a^ are peptides derived from a parent peptide, made by L-amino acids, in which the sequence is reversed and made by D-amino acids. This rearrangement combines the properties of D-peptides, namely protease-resistance, and with the reversed amino acid sequence leads to an imperfect topology overlapping with that of the parent peptide but achieve good mimicry for short sequences^12,13^.

We have applied this previously described strategy to a family of peptide BBB shuttles (H_2_N-HAIYPRH-CONH_2_ and H_2_N-THRPPMWSPVWP-CONH_2_^14^, namely HAI and THR peptides, respectively). BBB shuttles are molecular entities of diverse origin, *e*.*g*. synthetic or natural peptides, which have the capacity to cross the blood-brain barrier (BBB) and, when covalently conjugated to drugs unable to cross the BBB unaided, can deliver them into the central nervous system (CNS)^15^. Those that cross through receptor-mediated transcytosis are also included in the term ‘molecular Trojan horses’^16,17^. Although only a few clinical trials on BBB shuttles have been reported^18,19^, a huge number of these molecules effectively achieve their aim^20,21^. While parent versions of both HAI and THR displayed efficient BBB permeability in several studies, our laboratory demonstrated the BBB shuttle capability and protease-resistance of *retro*-D-THR^21^ and *retro*-D-HAI.

**Figure 1:**
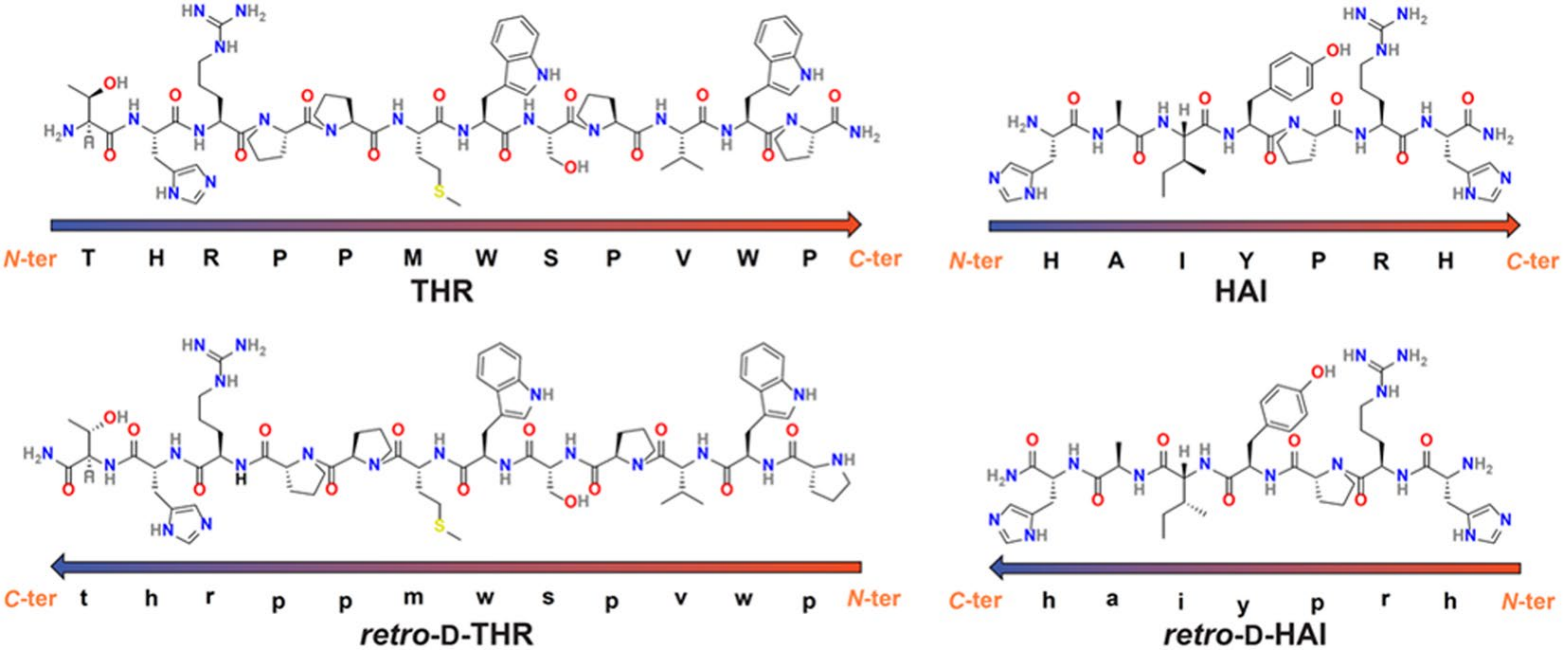
Peptide sequences of HAI, THR and the respective *retro*-D-versions.

Here we conducted immunogenicity studies for several peptide therapeutic candidates–two versions of HAI and THR peptides (Fig. 1), namely the parent and the respective *retro*-D-peptide.

## Results

### Validation of the *retro-*D-approach for HAI and THR – A structural perspective

**Figure 2:**
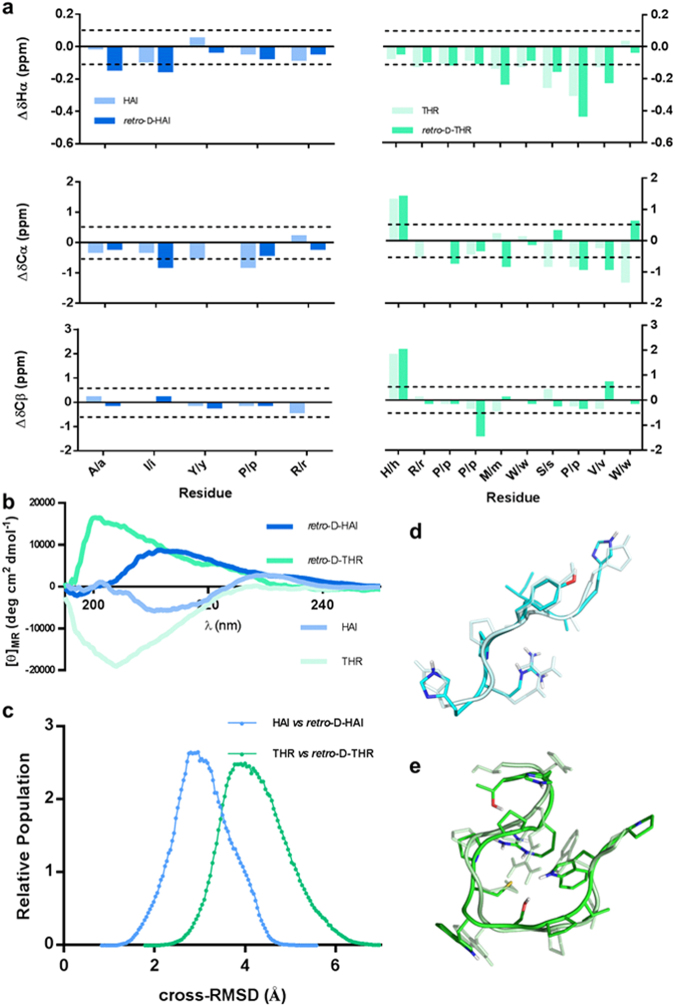
Structural analysis of the four peptides: (**a**) histograms showing the ^1^H_α_, ^13^C_α_ and ^13^C_β_ chemical shift deviations (CSD) from random coil (RC) of the major species (of HAI (left) and THR (right), and their respective *retro*-D-versions, aligned by amino acid type), (**b**) the circular dichroism (CD) spectra, (**c**) the cross-RMSD derived from the comparison of the whole REMD structure sets of parent and *retro*-D-version peptides, and the three-dimensional superposition of one pairing obtained from the cross-RMSD matrix of (**d**) HAI and *retro*-D-HAI, and of (**e**) THR and *retro*-D-THR.

The structural conformational arrangements of parent peptides and their respective *retro*-D-versions were studied by circular dichroism (CD) (Fig. 2b) and nuclear magnetic resonance (NMR) spectroscopy (Fig. 2a). While the coarse grain analysis by CD is consistent with similar conformational arrangements but not topologically exact, NMR adds further precision and shows how these peptides (both for HAI/*retro*-D-HAI and THR/*retro*-D-THR pairs) comprise several sets of thermodynamically stable conformational arrangements (see NMR data in Supporting Information; analysis of NMR conformationally sensitive parameters such as chemical shift deviations (CSD) from random coil (RC)^22^, coupling constants, NH temperature coefficients and NOE pattern reveal analogous results for the L-peptides and their *retro*-D-versions, thereby suggesting that they have similar conformational preferences). These results were contrasted by replica exchange molecular dynamics (REMD; Fig. 2c) and the overlapping of the two isomers was further analyzed. REMD allowed us to determine how these peptides are topologically disposed (see Computational Analysis in Supporting Information). Our results show that the parent and the *retro*-D-version of the same peptide can adopt a similar three-dimensional arrangement (Fig. 2c–e), in agreement with CD and NMR data.

### Study of humoral responses elicited by peptides made by L-amino acids and their respective *retro*-D-versions – Implications in therapy

To date, several studies have used D-peptides as tools for vaccine development. In these cases the peptides have been conjugated to large supramolecular entities, *e*.*g*. KLH and small unilamelar liposomes containing monophosphoryl lipid A^23,24^. Nevertheless, the use of *retro*-D-peptides as therapeutics was controversial twenty years ago^25,26^. Thus, to shed light on the immunogenicity of D-peptides and add further value to these compounds, we evaluated the immunological responses activated by *retro*-D-THR and *retro*-D-HAI peptides and compared them to that of their parent peptides. For this purpose, peptides (not conjugated) were *i*.*p*. administered to mice, and antibody titration was used as parameter to evaluate immunological (humoral) response by ELISA.

**Figure 3:**
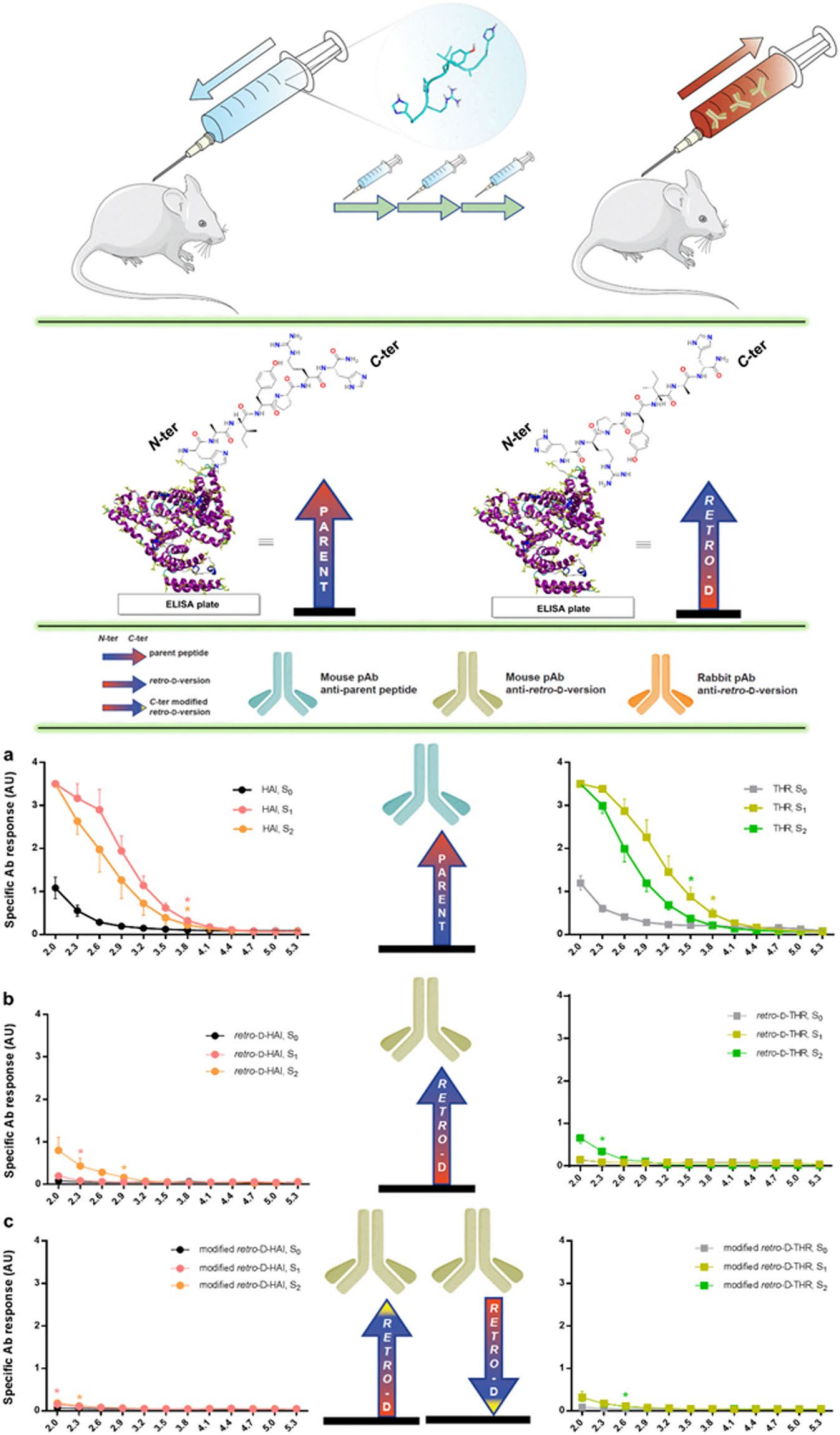
Titration of humoral response in mice by ELISA: (header) scheme of mice immunizations (shown with *retro*-D-HAI), ELISA evaluation of humoral response with peptide-BSA conjugates (HAI and *retro*-D-HAI shown), and the legend; (**a**) serum anti-parent peptides (HAI, THR), serum anti-*retro*-D-versions evaluated either with (**b**) *C*- or (**c**) *N*-terminus-exposed peptides. Initial consecutive significant differences between S_0_ with S_1_ or S_2_ are labeled by asterisk/s (no asterisk, no significant differences in the whole dilution interval).

Unconjugated L-versions of both peptides displayed a moderate immunogenicity response (Fig. 3a). Antibody titration of the first bleed showed a slightly higher signal for both peptides compared to the last bleeding. This signal was probably induced by the transition from IgM to IgG production in B cells. Thus, antibody titration decay is caused by a decrease in number, which is compensated by an increase in affinity and selectivity. In contrast, in the case of *retro*-D-peptides almost no signal was detected for specific antibodies (Fig. 3b). Thus, *retro*-D-versions appear to be much less immunogenic than their corresponding parent peptides.

Nevertheless, in order to discard the possibility that antibody recognition was dependent on the terminus exposed in these cases (since *retro*-D-versions have them inverted and are immobilized in the ELISA plate using the other terminus – see Fig. 1), we tested the serum of mice immunized against *retro*-D-peptides by attaching these peptides to the other terminus on the ELISA plate (Fig. 3c and Supplementary Fig. 1). In this regard, we evaluated the response of antibodies to the same peptide sequence but exposing the other terminus: the inversed order in *retro*-D-peptides (comparing with parent peptides) was corrected by displaying the opposite terminus. As an example, the HAI peptide attached to BSA (and subsequently to the plate) by the *N*-terminus displayed the sequence H_2_NOC-H ← R ← P ← Y ← […]-BSA-plate, and the peptide *retro*-D-HAI exposing the *N*-terminus showed H_2_N-h → r → p → y → […]-BSA-plate (see header of Fig. 3); compare Fig. 3a and c, and see Fig. 1 and Supplementary Fig. 6b. Similar results were obtained as when *retro*-D-peptides exposed the *C*-terminus (Fig. 3c), thereby confirming our hypothesis (antibody reactivity does not depend on the peptide termini used to immobilize them in the ELISA plate).

To the best of our knowledge, in all those immunological responses reported in the literature for either a *retro*-D-peptide or D-peptide (especially concerning vaccines), the parent one was derived from an existing sequence or even an immunodominant region of a viral protein^23,24^. Thus, these D- or *retro*-D-versions could have generated a rapid and huge immune response when administered to animals (*e*.*g*. serum IgG and other neutralizing factors against adeno-associated virus are highly prevalent in the healthy population^27^). In addition, a report describing D-proteins as entities exerting low immunogenicity was published 20 years ago^28^. We therefore hypothesized that *retro*-D-peptides with a parent sequence not related to any existing protein would not be immunogenic *per se*. To exclude this possibility in our study, we used a sequence similarity search tool (NCBI BLAST) to identify analogies between the parent peptides used and the whole data set of known peptide and protein sequences. No positive result was obtained, as previously described^14^.

Although, as shown above, *retro*-D-HAI and *retro*-D-THR are not immunogenic *per se*, we hypothesize that they could elicit an immunological response when conjugated to an immunogenic molecule. Thus, we conjugated these peptides to KLH and *s*.*c*. injected into rabbits. After the immunization, sera were tested by ELISA to identify antibodies displaying specific-peptide response. A strong increase in humoral response (see Supplementary Fig. 7) to these peptides was observed when compared to when they were used as unconjugated immunogens (Fig. 3b). The peptide-specific antibodies generated for both *retro*-D-peptides seems to be an immunoresponse facilitated by the anchoring molecule (KHL in this case). Hence, in order to be able to use this class of peptide (*retro*-D) for vaccine development, they should be coupled to other immunogenic structures to facilitate recognition by the immune system. However, if these peptides are to have applications as other types of therapeutic agent (and an eventual immune response is undesirable), they must be used (1) as a single therapeutic, (2) attached directly (2a) to the therapeutic agent or (2b) to other non-immunogenic structures.

## Discussion

The B cell response (antibody production) in mice immunized with D-peptides (precisely, *retro*-D) that were not coupled to any molecule was markedly lower compared to those immunized with the L-versions. We postulate that both versions of the peptide may eventually be recognized by a B Cell Receptor (BCR/membrane antibody); nevertheless, the D-version cannot be processed by peptidases and much less efficiently presented by the MCH-II to CD4+ T cells (T_H_) —the left-handed polyproline II extended conformation adopted by peptides when loaded in the antigen-binding groove of MCH-II is not accessible for D-peptides (thus hydrogen bonding between the amide bond of the peptide and the MHC-II is hindered, although binding pockets for the side chains are preserved). Therefore, the survival and proliferative signals to the B cells recognizing D-peptides are much lower compared to those that recognize L-peptides. Thus, we propose that D-peptides have two mechanisms by which to silence the immune system, namely protease-resistance—that hinders the processing by peptidases and thus their presentation by the MHC complexes in the right size^29–31^—and the conformational arrangement of the backbone, both of which are consequences of the inversed *α*-carbon configuration.

Since *retro*-D-peptides present good topological mimicry with their parent peptides, they are a simple solution for newly designed therapeutic peptides derived from diverse source of therapeutics with L-configuration, *e*.*g*. natural sources, phage display or computational design. Nevertheless, given the prior observations related to peptides composed by D-amino acids, we recommend a preliminary screening of the sequences of interest to test their prevalence and type of sources in nature in order to avoid eventual immune responses in advanced trials. In addition, we envisage the use of *retro*-D-peptides as protease-resistant variants to overcome immunological problems derived the intrinsic structure of drugs or vectors. As an example, viral vectors applied to gene therapy could be decorated with these peptides in order to allow them to escape from the immune system, either attached through a non-immunogenic linker or stand-alone (Supplementary Fig. 9), thereby avoiding systemic adverse reactions and neutralization by preexisting immunity^32^. Furthermore, their cell-tropism could be modulated (*e*.*g*. by using *retro*-D-versions of cell-penetrating peptides (CPP), blood-brain barrier shuttles (BBB shuttles), homing peptides (HP)), in order to target more effectively the diseased area and thus allowing the use of lower viral loads (*i*.*e*. reducing side effects and production costs).

## Methods

### Solid-Phase Peptide Synthesis, Cleavage and Work-Up

Peptides were manually synthesized as *C*-terminal amide on H-Rink^33^ amide-ChemMatrix (PCAS BioMatrix Inc.) using Fmoc/*t*Bu strategy. *N*-protected Fmoc-amino acids (4 eq.) were activated by Cl-HOBt^34^ (4 eq.) with *N*,*N*′-diisopropylcarbodiimide (DIPCDI) (4 eq.). The extent of the coupling reaction was monitored by the appropriate colorimetric test (ninhydrin^35^/chloranil^36^ for primary/secondary amine, respectively). The growing peptide chain was deprotected with 20% piperidine in DMF. Peptides were cleaved from the resin, and the side chains deprotected using TFA/TIS/H_2_O 95:2.5:2.5. After drying the cleavage residue using a nitrogen flow, three cycles of washes with cold methyl *tert*-butyl ether (MTBE), followed by centrifugation, were performed to precipitate the unprotected peptide.

### Peptide Purification and Characterization

Peptides were purified by reversed-phase HPLC using a symmetry C_18_ column (150 × 10 mm × 5 µm, 100 Å, Waters); solvents: H_2_O (0.1% TFA) and CH_3_CN (0.1% TFA); and flow rate of 3 mL/min. Purity of the peptides were assessed by analytical RP-HPLC (symmetry C_18_ column (150 × 4.6 mm × 5 µm, 100 Å, Waters); solvents: H_2_O (0.045% TFA) and CH_3_CN (0.036% TFA); flow rate of 1 mL/min) (Supplementary Fig. 2) and the identity was confirmed by MALDI-TOF MS (4700 MALDI-TOF spectrometer (PE Applied Biosystems), using an ACH matrix) and HRMS (Synapt HDMS (Waters) and LTQ-FT Ultra (Thermo Scientific)) (Supplementary Table 1).

### Structural Characterization of Peptides

Circular dichroism spectra (Fig. 2b) were obtained with a J-715 circular dichroism spectropolarimeter (Jasco). Parameters used: sensitivity (standard (100 mdeg)), λ start (250 nm), λ end (190 nm), data pitch (0.1 nm), scanning mode (continuous), scanning speed (10 nm/min), response (4 sec), band width (1.0 nm), and accumulation (3). NMR experiments were performed on a Bruker Avance III 600 MHz spectrometer equipped with a TCI cryoprobe. Samples were prepared by dissolving peptides in 50 mM NaCl, 25 mM sodium phosphate buffer, H_2_O/D_2_O 90:10, pH 7.4 at 5 mM, containing 0.02% NaN_3_. Chemical shifts were referenced to internal sodium-3-(trimethylsilyl)propanesulfonate (DSS). Water signal suppression was achieved by excitation sculpting^37^. Residue specific assignments were obtained from 2D total correlated spectroscopy (TOCSY) and correlation spectroscopy (COSY) experiments, while 2D nuclear Overhauser effect spectroscopy (NOESY) permitted sequence specific assignments. ^13^C resonances were assigned from 2D ^1^H-^13^C HSQC spectra. All experiments were performed at 298 K, except NOESY spectra, which were acquired at 278 K. Amide proton temperature coefficients were determined from a series of one-dimensional spectra acquired between 278 and 308 K (Supplementary Fig. 4, Tables 2–4). TOCSY and NOESY mixing times were 70 and 250 ms, respectively. Relative populations of the *cis*/*trans* isomers were estimated from integration of amide protons in the 1D ^1^H-NMR spectra or alternatively, when ^1^H integration was precluded by signal overlap, from the relative intensities of ^1^H-^13^C-HSQC crosspeaks corresponding to the Pro C_δ_ resonances. In those cases that ^1^H integration was possible, both methods provided identical results (Supplementary Table 5 and NMR data in Supporting Information).

### Computational Analysis of the Peptide Conformations

The preferential conformation adopted by each peptide system was evaluated by replica exchange molecular dynamics (REMD) simulations^38^. Simulations started from a linearly extended peptide conformation built with XLEaP program module from the AMBER14 molecular mechanics package^39^. The Amber ff99SB force field, together with the reoptimized proline omega-bond angle parameters^40^, was used. The initial extended peptide structure was first subjected to minimization protocol consisting of 1,000 steps of steep decent method followed by 500 steps of conjugate gradient method. Optimized structures were gradually heated to 300 K in 200 ps. The final state was chosen as the initial structure for all the 16 replicas. Temperatures were set in a range from 300 to 500 K^41^. Generalized Born model^42^ with an effective salt concentration of 0.2 M was deployed to mimic the solvation effect. Nonpolar solvation term was approximately represented by surface area term^43^. Integral time step was set to 1 fs. Temperature was regulated using Berendsen thermostat^44^ with a coupling time constant of 1 ps. SHAKE algorithm^45^ was used to constrain all the covalent bonds involving hydrogen atoms. Swaps between replicas were attempted every 2 ps, and ∼35% acceptance probability was obtained. Each replica was simulated during 150 ns. Snapshots were saved every 2 ps. To evaluate the degree of overlap between parent peptides and their *retro*-D-version forms, a non-symmetric RMSD distance matrix was built using the ptraj module of the Amber package. To preserve the correct alignment between the parent peptide and their *retro*-D-version, distances were computed between analog amino acids (*i*.*e*, the *N*-terminal amino acid of the parent peptide corresponds to *C*-terminal amino acid in its *retro*-D-version, and *vice versa*). The resulting RMSD matrix, composed of 1,000 equally spaced snapshots of the equilibrated part (100 ns) of the parent peptide and of its *retro*-D-version, was subjected to histogram analysis (Fig. 2c). R software^46^ was used in all statistical analyses. Additionally, 2D RMSD plots (mass weighted) for the same 1,000 equally spaced snapshots of each simulation were computed to visually determine the number of clusters visited by each peptide system during the replica exchange simulation (Supplementary Fig. 5). In order to further compute the similarity between the conformational space sampled by the trajectories of the parent peptide and *retro*-D-version, essential dynamics techniques^47,48^ (Supplementary Computation Analysis Section) were used.

### Peptide Conjugation to BSA or KLH

Each peptide (1 mg) was conjugated using either 1 mg of BSA ( ≥ 98%) or KLH (premium quality) obtained from Sigma-Aldrich. For each peptide sample, proteins were dissolved in 0.2 mL of MES buffer (0.1 M, pH 5) and 0.9 mg of EDC dissolved in 0.1 ml of MilliQ water was added (Supplementary Fig. 6b). After mixing for 10 min, 1 mg of the corresponding peptide dissolved in 0.55 mL of MES buffer was added and the mixture was left at room temperature for 3 h. Afterwards, it was filtered using Amicon Ultra-3K centrifugal filter devices at 5,000 rpm for 30 min, the residue was washed with MilliQ water and filtered again. Finally, this residue was dissolved in MilliQ water and freeze-dried in a vial for lyophilization. The lyophilized products were the corresponding conjugates. Peptide-BSA conjugates were identified through an UltrafleXtreme MALDI-TOF mass spectrometer (Bruker Daltonics), using a 2,6,-dihydroxyacetophenone (DHAP) matrix (Supplementary Fig. 6a).

### Mouse Immunization, Bleedings, and Serum Analysis by ELISA

Four groups of four BALB/c mice were treated with either one of the parent peptides or their *retro*-D-version. Each mouse received seven doses *i*.*p*. of 50 μg of peptide. Complete Freund’s^49^ adjuvant (CFA) was administered in the first dose, incomplete Freund’s adjuvant (IFA) in the subsequent five booster injections, and PBS in the last one. Bleedings for the titration of specific antibody production were performed before the first dose (time zero bleeding) and five days after the fourth and last dose (Supplementary Scheme 1). The peptide-specific humoral response was quantified by ELISA. MaxiSorp plates (Nunc) were treated with 0.1 mL of the corresponding peptide-BSA conjugate (1 μg) per well in carbonate buffer, overnight at 4 °C (Supplementary Fig. 6b). Afterwards, plates were blocked with 0.2 mL PBS-Tween 20 (T20) containing 2% of milk powder for 2 h a 37 °C. Each sample serum was consecutively diluted 1/2 (starting by dilution 1:100) in PBS-T20 and incubated for 1 h at 37 °C. Incubation with a secondary antibody (dil. 1/5,000) anti-Mouse IgG-HRP (ref. R1253HRP; batch 26922; Acris) was left for 1 h at 37 °C. Washes (x3) with PBST (300 μl/well) were applied after each antibody incubation. Finally, TMB (100 μl/well) was added and left for 30 min, when the stop solution (100 μl, HCl_aq_ 1 N) blocked the colorimetric reaction. Plates were read at 450 nm (Fig. 3a–c). All experiments were carried out following European guidelines (Directive 2010/63/EU, of 22 September 2010, on the protection of animals used for scientific purposes) and approved by the animal ethics committee of the Universitat Autònoma de Barcelona.

### Rabbit Immunization, Bleedings and Serum Analysis by ELISA

Each rabbit was immunized *s*.*c*. with five doses of 250 μg of the conjugate (peptide-KLH), each at different localization, altogether with CFA in the initial dose and IFA in the last four. Bleedings were obtained nine days after the third and the last dose (Supplementary Scheme 2). The peptide-specific humoral response was quantified by ELISA. Peptides were resuspended (8 mg/mL) in pre-adsorption buffer (23 mM NHS in DMF and 46 mM DCC in DMF, 1:1 (v/v)). MaxiSorp plates (Nunc) were treated with 0.1 mL of the corresponding peptide (1 μg), overnight at 4 °C. Afterwards, plates were blocked with 0.2 mL PBS-T20 containing 2% of milk powder for 2 h a 37 °C. Each sample serum was consecutively diluted 1/2 (starting by dilution 1:500) in PBS-T20 and incubated for 1 h at 37 °C. Then, an incubation with a secondary antibody (dil. 1/10,000) anti-Rabbit IgG- HRP (Ref. R1364HRP; batch 22489; Acris) was left for 1 h at 37 °C. Washes (x3) with PBST (300 μl/well) were applied after each antibody incubation. Finally, TMB (100 μl/well) was added and left for 30 min, when the stop solution (100 μl, HCl_aq_ 1 N) blocked the colorimetric reaction. Plates were read at 450 nm (Supplementary Fig. 7).

### Rabbit Polyclonal Antibodies Purification and Characterization

Half the serum samples were purified through an affinity column of protein A (HiTrap Protein A HP, GE Healthcare), dialyzed against PBS × 0.1 (membranes used: SnakeSkin® Dialysis Tubing, 10 K MWCO, 35 mm; Thermo Scientific) o/n at 4 °C with stirring at 100 rpm, and further characterized. Antibody purity was checked by SDS-PAGE (12% acrylamide gel; denaturing conditions (sample treated for 5 min at 100 °C in loading buffer 0.2 M DTT)), while ELISA was used to test the specificity of the response (Supplementary Fig. 8). ELISA was performed as before purification.

### Data Calculations and Statistics

Data were processed using GraphPad Prism 6.0 software. All results are presented as mean + SEM. Data from human serum stability assays were analyzed as non-linear one-phase exponential decay model for L-peptides and as standard linear model with zero slope for D-peptides. Deviations from a value of 100 at each dilution point were assessed using a linear model, in which group and interaction were included as covariates. In immune response studies, each dilution point (in ELISAs) was analyzed by a Mann-Whitney test to assess differences between S_0_ with S_1_ or S_2_. In both cases, 5% was set as a threshold for statistical significance.

### Data availability statement

All the relevant data supporting the findings are available from the corresponding author on reasonable request.

## Electronic supplementary material

Supplementary Information
