# Supplementary material for: Immunosilencing peptides by stereochemical inversion and sequence reversal: retro-D-peptides
Source: Sci Rep. 2018 Apr 24;8:6446. doi: 10.1038/s41598-018-24517-6 (PMC5915530; doi:10.1038/s41598-018-24517-6)
Supplement: Supplementary file 1 — Supplementary Information [file 41598_2018_24517_MOESM1_ESM.docx]

**Immunosilencing peptides by stereochemical inversion and sequence reversal: *retro*-D-peptides**

Pol Arranz-Gibert^1^, Sonia Ciudad^1^, Jesús Seco^1^, Jesús García^1^, Ernest Giralt^1,2,*^, Meritxell Teixidó^1,*^

**Author Affiliations:**

1) Institute for Research in Biomedicine (IRB Barcelona), Barcelona Institute of Science and Technology (BIST), Baldiri Reixac 10, Barcelona, E-08028, Spain.

2) Department of Organic Chemistry, University of Barcelona, Martí i Franquès 1-11, Barcelona, E-08028, Spain.

**^*^Corresponding Authors:**

E-mail: meritxell.teixido@irbbarcelona.org and ernest.giralt@irbbarcelona.org

**Table of Contents**

Materials and Methods S4

Peptide Characterization S5

Table 1 S5

Figure 1 S6

Figure 2 S7

Structural Analysis of the Peptides S8

NMR data S8

Figure 3a S10

Figure 3b S11

Figure 3c S12

Figure 3d S13

Table 2a S14

Table 2b S15

Table 3a S17

Table 3b S18

Table 4a S20

Table 4b S21

Table 5 S22

Computational Analysis S23

Figure 4 S24

Peptide-Protein Conjugate Characterization S25

Figure 5a S25

Figure 5b S26

Mouse Immunization Chronology S28

Scheme 1 S28

Rabbit Immunization Chronology S29

Scheme 2 S29

Immunological Study in Rabbit S30

Figure 6 S30

Rabbit Polyclonal Antibody Purification and Characterization S31

Figure 7 S31

Other Supplementary Information S32

Figure 8 S32

Abbreviations S33

References S35

**Materials and Methods**

Protected amino acids and resins were supplied by Luxembourg Industries (Tel-Aviv, Israel), Neosystem (Strasbourg, France), Calbiochem-Novabiochem AG (Laüfelfingen, Switzerland), PolyPeptide Laboratories (Torrance, CA USA), Bachem AG (Bubendorf, Switzerland), and Iris Biotech (Marktredwitz, Germany). Ninhydrin was from Fluka Chemika (Buchs, Switzerland). Solvents for peptide synthesis and RP-HPLC were from Scharlau or SDS (Barcelona, Spain). Trifluoroacetic acid was purchased from KaliChemie (Bad Wimpfen, Germany). The other chemicals used were supplied by Aldrich and were of the highest purity commercially available. NMR experiments were carried out on a Bruker Avance III 600 MHz spectrometer equipped with a TCI cryoprobe. Mass spectra were recorded on an Applied Biosystems 4700 MALDI-TOF mass spectrometer (PE Applied Biosystems, Foster City, CA USA), using an ACH matrix. Conjugates were analyzed through a UltrafleXtreme MALDI-TOF mass spectrometer (Bruker Daltonics, Billerica, MA United States), using a DHAP matrix. High-resolution mass spectra were recorded on a Synapt HDMS (Waters, Manchester, UK) and on a LTQ-FT Ultra (Thermo Scientific). RP-HPLC chromatograms were recorded on a Waters model Alliance 2695 with photodiode array (PDA) detector 996 from Waters (Waters, Milford, CT USA) using a Sunfire C_18_ column (150 × 4.6 mm × 5 µm, 100 Å, Waters); solvents: H_2_O (0.045% TFA) and CH_3_CN (0.036% TFA); flow rate of 1 mL/min; and software Millenium version 4.0. HPLC-MS [Waters model Alliance 2796, quaternary pump, Waters 2487 with UV/Vis dual absorbance detector, ESI-MS model Micromass ZQ and Masslynx version 4.0 software (Waters)] was done using a Sunfire 300 C_18_ column (150 × 3.9 mm × 5 µm, 300 Å, Waters); solvents: H_2_O (0.1% formic acid) and CH_3_CN (0.07% formic acid); and flow rate of 1 mL/min. The products were purified in a 2545 binary gradient module, a 2767 manager collector, and a 2998 photodiode array (PDA) detector (Waters), with Masslynx version 4.1 (Waters), and a Sunfire C_18_ column (150 × 10 mm × 5 µm, 100 Å, Waters); solvents: H_2_O (0.1% TFA) and CH_3_CN (0.1% TFA); and flow rate of 3 mL/min. Graphics were performed using GraphPad Prism version 6.01 for Windows (GraphPad Software, La Jolla, CA USA), where the following significance descriptors were used:

| **P value** | **Wording** | **Summary** |
| --- | --- | --- |
| < 0.0001 | Extremely significant | **** |
| 0.0001 to 0.001 | Extremely significant | *** |
| 0.001 to 0.01 | Very significant | ** |
| 0.01 to 0.05 | Significant | * |
| ≥ 0.05 | Not significant | ns |

**Peptide characterization**

| **Peptide ID/**  **Composition** | **MW**  **(g/mol)** | **HPLC t_R_**  **(min)** | **Purity***^a^*  **(%)** | **MALDI-TOF**  **[M+H]^+^** | **HRMS found**  **[M]** | **HRMS calcd.**  **[M]** |
| --- | --- | --- | --- | --- | --- | --- |
| HAI  C_41_ H_61_ O_8_ N_15_ | 892.0 | 3.26 | >99 | 892.6 | 891.48155 | 891.48275 |
| THR  C_71_ H_100_ O_14_ N_20_ S_1_ | 1489.8 | 4.36 | >99 | 1489.9 | 1488.74256 | 1488.74486 |
| *retro*-D-HAI  C_41_ H_61_ O_8_ N_15_ | 892.0 | 3.61 | >99 | 892.7 | 891.48244 | 891.48275 |
| *retro*-D-THR  C_71_ H_100_ O_14_ N_20_ S_1_ | 1489.8 | 4.50 | >99 | 1489.9 | 1488.74466 | 1488.74486 |
| *retro*-D-HAI-Dpr  C_44_ H_67_ O_9_ N_17_ | 978.1 | 3.20 | >99 | 978.5 | 977.52875 | 977.53077 |
| *retro*-D-THR-Dpr  C_74_ H_106_ O_15_ N_22_ S_1_ | 1575.9 | 4.07 | >99 | 1575.9 | 1574.79039 | 1574.79287 |

*^a^*After purification by RP-HPLC.

**Table 1.** Characterization of the set of peptides studied by RP-HPLC at 220 nm (gradient from 0 to 100% CH_3_CN in 8 min; Sunfire C_18_ column), MALDI-TOF MS, and HRMS.

**Figure 1.** Peptide sequences of *retro*-D-versions containing the diaminopropionic acid (Dpr) residue at the *C*-terminus.

**Figure 2.** RP-HPLC chromatograms (gradient from 0 to 100% CH_3_CN in 8 min) of the pure peptides: (**a**) HAI, (**b**) THR, (**c**) *retro*-D-HAI, (**d**) *retro*-D-THR, (**e**) *retro*-D-HAI-Dpr, and (**f**) *retro*-D-THR-Dpr.

**Structural Analysis of the Peptides**

**NMR data**

HAI and *retro*-D-HAI peptides

Peptides HAI and *retro*-D-HAI were studied in aqueous solution by means of NMR. Two sets of signals were present in the spectra of both peptides. In both cases, the *trans* conformer of the Xaa-Pro amide bond was confirmed as the major species, as determined on the basis of the presence of strong sequential d_αδ_ NOE (Tyr4/Pro5 for HAI and Arg2/Pro3 for *retro*-D-HAI)[^1^](#_ENREF_1), as well as on the ^13^C_β_ - ^13^C_γ_ chemical shift differences observed for the Pro residues (Δδ = 4.7 and 4.8 ppm for HAI and *retro*-D-HAI, respectively)[^2^](#_ENREF_2). The *cis* Xaa-Pro conformers were assigned by their characteristic d_αα_ NOE cross-peaks (Tyr4/Pro5 for HAI or Arg2/Pro3 for *retro*-D-HAI) and by the Pro ^13^C_β_ - ^13^C_γ_ chemical shift difference (Δδ = 9.4 and 9.9 ppm for HAI and *retro*-D-HAI, respectively), and they accounted for 24% in HAI and 10% in *retro*-D-HAI (see **Supplementary Table 2a** and **Table 5**).

The major *trans* conformers of HAI and *retro*-D-HAI had very similar proton (^1^H_α_) and carbon (^13^C_α_ and ^13^C_β_) chemical shifts when compared by residue type (**Fig. 2a**). These NMR chemical shifts depend on the backbone conformation and are sensitive to local structure, thereby suggesting that both peptides have similar conformational preferences. Comparison of observed and random coil (RC) ^1^H_α_, ^13^C_β_ and ^13^C_α_ chemical shifts[^3^](#_ENREF_3) indicated a mainly RC state for these peptides. The low dispersion of NH amide proton signals (**Supplementary Fig. 3b**) and the absence of inter-residue non-sequential NOEs provided further evidence of flexibility. In addition, the ^3^J_αNH_ couplings and amide NH temperature coefficients observed for these peptides were within the range expected for RC conformations (**Supplementary Table 4b**).

The *cis* conformers of HAI and *retro*-D-HAI were partially assigned due to their lower signal intensity and to the spectral overlap. The NH chemical shift dispersion of the two *cis* conformers was again consistent with RC conformations (**Supplementary Fig. 4b**). When measurable, these conformers showed coupling constants between 6.7 and 8.0, thereby indicating a disordered structure (**Supplementary Table 4a**).

In summary, it can be concluded that the L-peptide and its *retro*-D-version exist in a flexible conformation rather than in a well-defined secondary structure.

THR and *retro*-D-THR peptides

A subset of minor NMR signals coexisting with those of the major species was present in the spectra of THR and *retro*-D-THR peptides in aqueous solution. The major species of THR (~70%) and *retro*-D-THR (at least 90%) were found to contain all Xaa-Pro bonds in *trans* conformation, as deduced from the presence of strong sequential d_αδ_ NOEs (Arg3/Pro4, Pro4/Pro5, Ser8/Pro9 and Trp11/Pro12 for THR, and Val3/Pro4, Met7/Pro8, Pro8/Pro9 for *retro*-D-THR) and Pro ^13^C_β_ - ^13^C_γ_ values (3.5, 4.5, 4.6 and 4.9 ppm for THR and 4.9, 3.5 and 4.7 for *retro*-D-THR). The minor species of THR and *retro*-D-THR could not be assigned due to low intensity of the signal and overall spectra complexity.

The low dispersion of the amide NH protons in the THR and *retro*-D-THR major species, with the NH appearing between 7.62 and 8.36 ppm in THR and between 7.60 and 8.40 ppm in *retro*-D-THR, suggests a flexible conformation (**Supplementary Fig. 3c**). Deviations of the ^1^H_α_, ^13^C_β_ and ^13^C_α_ chemical shifts from RC[^3^](#_ENREF_3) values revealed that the two peptides have a similar pattern of secondary chemical shifts when compared by amino acid equivalence (pairing *N*- to *C*-ter; **Fig. 2a**), thereby suggesting that the peptide backbone of the major all *trans* conformer takes on very similar conformations. In contrast, similarity was highly reduced when comparing by sequence order (*N*- to *N*- and *C*- to *C*-ter; **Supplementary Fig. 3d**). A series of residues simultaneously exhibiting a moderate ^1^H_α_ and ^13^C_α_ upfield shift was observed. This trend does not correspond to any of the major well-known secondary structural motifs (α-helix and β-sheet) but may reflect that some parts of the peptide tend to be more conformationally restrained. However, we did not observe any non-sequential NOE characteristic of local structure. The ^3^J_αNH_ couplings were between 6 and 8 ppm, thereby suggesting either a RC state or fast equilibrium between multiple conformational states (**Supplementary Table 4b**). Amide NH protons displayed large negative temperature coefficient values, typical of solvent-accesible NH protons (**Supplementary Table 4b**).

**Figure 3a.** ^1^H-NMR spectra of HAI, THR and their respective *retro*-D-versions.

**Figure 3b**. Downfield region of ^1^H-NMR spectra of HAI (bottom) and its respective *retro*-D-version (top), acquired at 278K.

**Figure 3c.** Downfield region of ^1^H-NMR spectra of THR (bottom) and its respective *retro*-D-version (top), acquired at 278K.

**
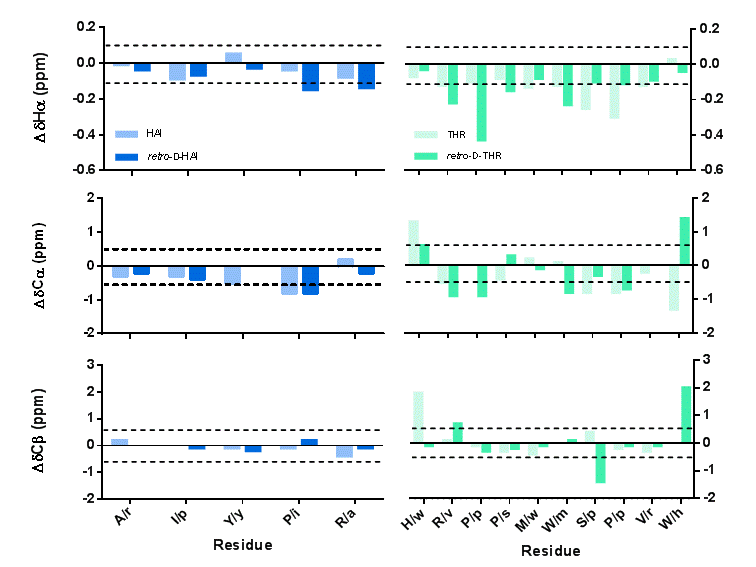
**

**Figure 3d.** Histograms showing the ^1^H_α_, ^13^C_α_ and ^13^C_β_ chemical shift deviations (CSD) from RC[^3^](#_ENREF_3) values for the major species of HAI (left) and THR (right), and their respective *retro*-D-versions, aligned by sequence order.

**Table 2a.** ^1^H-NMR chemical shifts (ppm) of HAI and *retro-*D-HAI peptides.

| nº | Residue | NH | | |  | C_α_H | | |  | C_β_H | | |
| --- | --- | --- | --- | --- | --- | --- | --- | --- | --- | --- | --- | --- |
|  |  | HAI  (*cis*) | HAI  (*trans*) | *retro-*D * |  | HAI  (*cis*) | HAI  (*trans*) | *retro-*D * |  | HAI  (*cis*) | HAI  (*trans*) | *retro-*D * |
|  |  |  |  |  |  |  |  |  |  |  |  |  |
| 1 | His/D-His | - | - | - |  | 3.90 | 4.02 | 4.00 |  | 3.13 | 3.13 | 3.07 |
|  |  |  |  |  |  |  |  |  |  |  | 3.06 | 3.02 |
| 2 | Ala/D-Arg | ** | ** | ** |  | 4.36 | 4.31 | 4.61 |  | 1.34 | 1.28 | 1.78 |
|  |  |  |  |  |  |  |  |  |  |  |  | 1.69 |
| 3 | Ile/D-Pro | 8.21 | 8.18 | - |  | 4.11 | 4.08 | 4.38 |  | 1.76 | 1.71 | 2.25 |
|  |  |  |  |  |  |  |  |  |  |  |  | 1.82 |
| 4 | Tyr/D-Tyr | 8.07 | 8.26 | 8.27 |  | 4.49 | 4.89 | 4.52 |  | 2.88 | 3.07 | 3.02 |
|  |  |  |  |  |  |  |  |  |  |  | 2.8 | 2.94 |
| 5 | Pro/D-Ile | - | - | 7.86 |  | 3.93 | 4.39 | 4.02 |  | 1.93 | 2.24 | 1.67 |
|  |  |  |  |  |  |  |  |  |  | 1.81 | 1.82 |  |
| 6 | Arg/D-Ala | 8.59 | 8.39 | 8.24 |  | 4.20 | 4.26 | 4.18 |  | 1.75 | 1.76 | 1.33 |
|  |  |  |  |  |  |  |  |  |  |  |  |  |
| 7 | His/D-His | ** | 8.30 | 8.17 |  | 4.59 | 4.62 | 4.55 |  | 3.08 | 3.15 | 3.11 |
|  |  |  |  |  |  |  |  |  |  |  | 3.10 | 3.08 |
|  |  |  |  |  |  |  |  |  |  |  |  |  |

* *Complete assignment of the cis conformer was not possible.*

** *The fact that these NH amide protons are not observed can be due to a major flexibility in both the C- and N-terminal region (protons highly exposed to the solvent).*

(continued from the previous table)

| nº | Residue | others | | |
| --- | --- | --- | --- | --- |
|  |  | HAI (*cis*) | HAI (*trans*) | *retro-*D |
|  |  |  |  |  |
| 1 | His/D-His | H_2_ 7.92 | H_2_ 7.91 | H_2_ 7.87 |
|  |  | H_4_ 7.07 | H_4_ 7.07 | H_4_ 7.03 |
| 2 | Ala/D-Arg |  |  | CγH_2_ 1.63 |
|  |  |  |  | CδH_2_ 3.20 |
| 3 | Ile/D-Pro | CγH_2_ 1.13, 1.40 | CγH_2_ 1.08, 1.35 | CγH_2_ 1.98 |
|  |  | CγH_3_ 0.82 | CγH_3_ 0.77 | CδH_2_ 3.71, 3.61 |
|  |  | CδH_3_ 0.83 | CδH_3_ 0.80 |  |
| 4 | Tyr/D-Tyr | H_2,6_ 7.11 | H_2,6_ 7.14 | H_2,6_ 7.10 |
|  |  | H_3,5_ 6.84 | H_3,5_ 6.80 | H_3,5_ 6.80 |
| 5 | Pro/D-Ile | CγH_2_ 1.74, 1.68 | CγH_2_ 1.98 | CγH_2_ 1.36, 1.06 |
|  |  | CδH_2_ 3.48, 3.39 | CδH_2_ 3.59, 3.79 | CγH_3_ 0.76 |
|  |  |  |  | CδH_3_ 0.80 |
| 6 | Arg/D-Ala | CγH_2_ 1.57 | CγH_2_ 1.59 |  |
|  |  | CδH_2_ 3.17 | CδH_2_ 3.19 |  |
| 7 | His/D-His | H_2_ 7.90 | H_2_ 7.91 | H_2_ 7.91 |
|  |  | H_4_ 7.03 | H_4_ 7.06 | H_4_ 7.05 |
|  |  |  |  |  |

**Table 2b.** ^1^H-NMR chemical shifts (ppm) of THR and *retro-*D-THR peptides.

| nº | Residue | NH | |  | C_α_H | |  | C_β_H | |
| --- | --- | --- | --- | --- | --- | --- | --- | --- | --- |
|  |  | THR* | *retro-*D * |  | THR* | *retro-*D * |  | THR* | *retro-*D * |
|  |  |  |  |  |  |  |  |  |  |
| 1 | Thr/D-Pro | - | - |  | 3.63 | 4.36 |  | 4.03 | 2.44 |
|  |  |  |  |  |  |  |  |  | 2.02 |
| 2 | His/D-Trp | ** | 7.60 |  | 4.66 | 4.63 |  | 3.10 | 3.21 |
|  |  |  |  |  |  |  |  | 3.07 |  |
| 3 | Arg/D-Val | 8.36 | 7.74 |  | 4.53 | 4.22 |  | 1.70 | 1.85 |
|  |  |  |  |  |  |  |  | 1.62 |  |
| 4 | Pro/D-Pro | - | - |  | 4.63 | 3.99 |  | 2.34 | 2.05 |
|  |  |  |  |  |  |  |  | 1.89 | 1.51 |
| 5 | Pro/D-Ser | - | 8.20 |  | 4.36 | 4.32 |  | 2.21 | 3.87 |
|  |  |  |  |  |  |  |  | 1.73 | 3.85 |
| 6 | Met/D-Trp | 8.32 | 7.91 |  | 4.35 | 4.58 |  | 2.00 | 3.20 |
|  |  |  |  |  |  |  |  | 1.96 |  |
| 7 | Trp/D-Met | 8.02 | 7.63 |  | 4.54 | 4.59 |  | 3.23 | 1.82 |
|  |  |  |  |  |  |  |  |  | 1.65 |
| 8 | Ser/D-Pro | 7.62 | - |  | 4.53 | 4.33 |  | 3.61 | 2.31 |
|  |  |  |  |  |  |  |  |  | 1.82 |
| 9 | Pro/D-Pro | - | - |  | 4.16 | 4.33 |  | 2.14 | 2.25 |
|  |  |  |  |  |  |  |  | 1.68 | 1.82 |
| 10 | Val/D-Arg | 7.79 | 8.36 |  | 4.00 | 4.25 |  | 1.94 | 1.75 |
|  |  |  |  |  |  |  |  |  | 1.71 |
| 11 | Trp/D-His | 8.2 | 8.40 |  | 5.02 | 4.69 |  | 3.30 | 3.11 |
|  |  |  |  |  |  |  |  | 3.14 |  |
| 12 | Pro/D-Thr | - | 8.07 |  | 4.35 | 4.27 |  | 2.22 | 4.22 |
|  |  |  |  |  |  |  |  |  |  |

* *For the both peptides, complete assignment of the cis conformer was not possible due to overlapping*

** *The fact that this NH amide proton is not observed can be due to a major flexibility in the N-terminal region, which greatly exposes this proton to the solvent.*

(continued from the previous table)

| nº | Residue | others | |
| --- | --- | --- | --- |
|  |  | THR* | *retro-*D * |
| 1 | Thr/D-Pro | CγH_3_ 1.21 | CγH_2_ 2.03, 1.95 |
|  |  |  | CδH_2_ 3.36 |
| 2 | His/D-Trp | H_2_ 7.89 | NH 10.12 |
|  |  | H_4_ 7.02 | H_2_ 7.24 |
|  |  |  | H_4_ 7.47 |
|  |  |  | H_5_ 7.26 |
|  |  |  | H_6_ 7.12 |
|  |  |  | H_7_ 7.50 |
| 3 | Arg/D-Val | CγH_2_ 1.51 | CγH_3_ 0.83, 0.76 |
|  |  | CδH_2_ 3.02, 2.95 |  |
| 4 | Pro/D-Pro | CγH_2_ 1.99 | CγH2 1.79 |
|  |  | CδH_2_ 3.67, 3.49 | CδH_2_ 3.45, 3.34 |
| 5 | Pro/D-Ser | CγH_2_ 1.98 | - |
|  |  | CδH_2_ 3.78, 3.63 |  |
| 6 | Met/D-Trp | CγH_2_ 2.51, 2.44 | NH 10.17 |
|  |  | CεH_3_ 2.07 | H_2_ 7.23 |
|  |  |  | H_4_ 7.54 |
|  |  |  | H_5_ 7.26 |
|  |  |  | H_6_ 7.12 |
|  |  |  | H_7_ 7.50 |
| 7 | Trp/D-Met | NH 10.13 | CγH_2_ 2.34, 2.30 |
|  |  | H_2_ 7.22 | εCH_3_ 2.03 |
|  |  | H_4_ 7.53 |  |
|  |  | H_5_ 7.17 |  |
|  |  | H_6_ 7.12 |  |
|  |  | H_7_ 7.47 |  |
| 8 | Ser/D-Pro | - | CγH_2_ 1.96 |
|  |  |  | CδH_2_ 3.56, 3.50 |
| 9 | Pro/D-Pro | CγH_2_ 1.90 | CγH_2_ 2.04 |
|  |  | CδH_2_ 3.55, 3.39 | CδH_2_ 3.76, 3.65 |
| 10 | Val/D-Arg | CγH_3_ 0.84, 0.79 | CγH_2_ 1.56 |
|  |  |  | CδH_2_ 3.15 |
| 11 | Trp/D-His | NH 10.09 | H_2_ 7.82 |
|  |  | H_2_ 7.23 | H_4_ 7.00 |
|  |  | H_4_ 7.69 |  |
|  |  | H_5_ 7.17 |  |
|  |  | H_6_ 7.12 |  |
|  |  | H_7_ 7.49 |  |
| 12 | Pro/D-Thr | CγH_2_ 1.90 | CγH_3_ 1.15 |
|  |  | CδH_2_ 3.78, 3.42 |  |
|  |  |  |  |

**Table 3a.** ^13^C-NMR chemical shifts (ppm) of HAI and *retro-*D-HAI peptides.

| nº | Residue |  | C_α_ | | |  | C_β_ | | |
| --- | --- | --- | --- | --- | --- | --- | --- | --- | --- |
|  |  |  | HAI (*cis*) | HAI (*trans*) | *retro-*D * |  | HAI (*cis*) | HAI (*trans*) | *retro-*D * |
|  |  |  |  |  |  |  |  |  |  |
| 1 | His/D-His |  | 56.0 | 56.1 | 56.3 |  | 31.4 | 32.2 | 32.6 |
| 2 | Ala/D-Arg |  | 52.2 | 52.2 | 53.8 |  | 19.4 | 19.3 | 30.2 |
| 3 | Ile/D-Pro |  | 61.1 | 60.8 | 63.1 |  | 38.9 | 38.8 | 32.0 |
| 4 | Tyr/D-Tyr |  | 55.7 | 55.3 | 57.9 |  | 40.5 | 38.2 | 38.6 |
| 5 | Pro/D-Ile |  | 62.6 | 63.0 | 60.3 |  | 34.1 | 32.0 | 39.0 |
| 6 | Arg/D-Ala |  | 56.4 | 56.2 | 52.3 |  | 30.7 | 30.5 | 19.0 |
| 7 | His/D-His |  | 55.8 | 55.8 | 56.0 |  | 31.5 | 30.9 | 30.8 |

* *Complete assignment of the cis conformer was not possible.*

(continued from the previous table)

| nº | Residue | others | | |
| --- | --- | --- | --- | --- |
|  |  | HAI (*cis*) | HAI (*trans*) | *retro-*D |
|  |  |  |  |  |
| 1 | His/D-His | H_2_ 138.5 | H_2_ 138.3 | H_2_ 138.5 |
|  |  | H_4_ 120.2 | H_4_ 120.2 | H_4_ 120.3 |
| 2 | Ala/D-Arg |  |  | CγH_2_ 26.7 |
|  |  |  |  | CδH_2_ 43.4 |
| 3 | Ile/D-Pro | CγH_2_ 27.2 | CγH_2_ 27.1 | CγH_2_ 27.2 |
|  |  | CγH_3_ 17.3 | CγH_3_ 17.4 | CδH_2_ 50.6 |
|  |  | CδH_3_ 12.8 | CδH_3_ 12.7 |  |
| 4 | Tyr/D-Tyr | H_2,6_ 133.2 | H_2,6_ 133.3 | H_2,6_ 133.1 |
|  |  | H_3,5_ 118.4 | H_3,5_ 118.1 | H_3,5_ 118.1 |
| 5 | Pro/D-Ile | CγH_2_ 24.7 | CγH_2_ 27.3 | CγH_2_ 26.9 |
|  |  | CδH_2_ 49.9 | CδH_2_ 50.6 | CγH_3_ 17.1 |
|  |  |  |  | CδH_3_ 12.6 |
| 6 | Arg/D-Ala | CγH_2_ 27.1 | CγH_2_ 27.1 |  |
|  |  | CδH_2_ 43.3 | CδH_2_ 43.3 |  |
| 7 | His/D-His | H_2_ 138.2 | H_2_ 138.3 | H_2_ 138.1 |
|  |  | H_4_ 119.7 | H_4_ 119.9 | H_4_ 119.9 |

**Table 3b.** ^13^C-NMR chemical shifts (ppm) of THR and *retro-*D-THR peptides.

| nº | Residue |  | C_α_ | |  | C_β_ | |
| --- | --- | --- | --- | --- | --- | --- | --- |
|  |  |  | THR* | *retro-*D * |  | THR* | *retro-*D * |
|  |  |  |  |  |  |  |  |
| 1 | Thr/D-Pro |  | 61.9 | 62.2 |  | 70.1 | 32.6 |
| 2 | His/D-Trp |  | 56.3 | 58.1 |  | 30.8 | 29.5 |
| 3 | Arg/D-Val |  | 53.5 | 58.9 |  | 30.3 | 33.3 |
| 4 | Pro/D-Pro |  | 61.5 | 62.8 |  | 30.8 | 31.8 |
| 5 | Pro/D-Ser |  | 63.1 | 58.6 |  | 31.8 | 63.6 |
| 6 | Met/D-Trp |  | 55.6 | 57.4 |  | 32.5 | 29.5 |
| 7 | Trp/D-Met |  | 57.6 | 52.5 |  | 29.6 | 33.0 |
| 8 | Ser/D-Pro |  | 55.6 | 61.2 |  | 63.7 | 30.7 |
| 9 | Pro/D-Pro |  | 63.0 | 62.8 |  | 31.9 | 32.0 |
| 10 | Val/D-Arg |  | 62.0 | 56.0 |  | 32.6 | 30.8 |
| 11 | Trp/D-His |  | 54.4 | 56.4 |  | 29.6 | 31.0 |
| 12 | Pro/D-Thr |  | 63.1 | 61.4 |  | 31.9 | 69.6 |
|  |  |  |  |  |  |  |  |

* *For both peptides, complete assignment of the cis conformer was not possible due to spectra complexity*

(continued from the previous table)

| nº | Residue | others | |
| --- | --- | --- | --- |
|  |  | THR * | *retro-*D * |
| 1 | Thr/D-Pro | CγH_3_ 21.2 | CγH_2_ 26.5 |
|  |  |  | CδH_2_ 49.3 |
| 2 | His/D-Trp | H_2_ 138.3 | H_2_ 127.2 |
|  |  | H_4_ 119.8 | H_4_ 120.8 |
|  |  |  | H_5_ 124.5 |
|  |  |  | H_6_ 121.9 |
|  |  |  | H_7_ 114.5 |
| 3 | Arg/D-Val | CγH_2_ 26.6 | CγH_3_ 21.0, 19.8 |
|  |  | CδH_2_ 43.3 |  |
| 4 | Pro/D-Pro | CγH_2_ 27.3 | CγH2 27.1 |
|  |  | CδH_2_ 50.5 | CδH_2_ 50.5 |
| 5 | Pro/D-Ser | CγH_2_ 27.3 | - |
|  |  | CδH_2_ 50.5 |  |
| 6 | Met/D-Trp | CγH_2_ 31.9 | H_2_ 127.2 |
|  |  | CεH_3_ 16.8 | H_4_ 120.8 |
|  |  |  | H_5_ 124.5 |
|  |  |  | H_6_ 121.9 |
|  |  |  | H_7_ 114.5 |
| 7 | Trp/D-Met | H_2_ 127.2 | CγH_2_ 31.7 |
|  |  | H_4_ 120.7 | CεH_3_ 16.9 |
|  |  | H_5_ 122.1 |  |
|  |  | H_6_ 121.9 |  |
|  |  | H_7_ 114.6 |  |
| 8 | Ser/D-Pro | - | CγH_2_ 27.2 |
|  |  |  | CδH_2_ 50.3 |
| 9 | Pro/D-Pro | CγH_2_ 27.3 | CγH_2_ 27.3 |
|  |  | CδH_2_ 50.4 | CδH_2_ 50.5 |
| 10 | Val/D-Arg | CγH_3_ 20.4, 21.0 | CγH_2_ 27.0 |
|  |  |  | CδH_2_ 43.3 |
| 11 | Trp/D-His | H_2_ 124.6 | H_2_ 138.5 |
|  |  | H_4_ 121.0 | H_4_ 119.8 |
|  |  | H_5_ 122.1 |  |
|  |  | H_6_ 121.9 |  |
|  |  | H_7_ 114.6 |  |
| 12 | Pro/D-Thr | CγH_2_ 27.1 | CγH_3_ 21.4 |
|  |  | CδH_2_ 50.7 |  |
|  |  |  |  |

**Table 4a**. ^3^J_αNH_ (Hz) and -Δδ/ΔT (ppb/K) of the HAI and *retro-*D-HAI peptides.

| nº | Residue | ^3^JαN (Hz) | | | -Δδ/ΔT (ppb/K) | | |
| --- | --- | --- | --- | --- | --- | --- | --- |
|  |  | HAI (*cis*) | HAI (*trans*) | *retro-*D | HAI (*cis*) | HAI (*trans*) | *retro-*D |
|  |  |  |  |  |  |  |  |
| 1 | His/D-His | - | - | - | - | - | - |
| 2 | Ala/D-Arg | *n.o.* | *n.o.* | *n.o.* | - | - | - |
| 3 | Ile/D-Pro | 7.8 | 8.5 | - | 10.0 | 10.0 | - |
| 4 | Tyr/D-Tyr | 8.0 | 8.0 | 6.7 | 8.5 | 10.5 | 11.5 |
| 5 | Pro/D-Ile | - | - | 8.8 | - | - | 7.0 |
| 6 | Arg/D-Ala | 6.7 | 6.9 | 5.8 | 8.5 | 9.0 | 8.0 |
| 7 | His/D-His | *n.o.* | *b.s.* | 6.1 | - | 11 | 11.0 |
|  |  |  |  |  |  |  |  |

*n.o. ≡ not observed*

*b.s. ≡ broad signal*

**Table 4b**. ^3^J_αNH_ (Hz) and -Δδ/ΔT (ppb/K) of the THR and *retro-*D-THR peptides.

| nº | Residue | ^3^JαN (Hz) | | -Δδ/ΔT (ppb/K) | |
| --- | --- | --- | --- | --- | --- |
|  |  | THR | *retro-*D | THR | *retro-*D |
|  |  |  |  |  |  |
| 1 | Thr/D-Pro | - | - | - | - |
| 2 | His/D-Trp | *n.o.* | *o.* | - | 6.0 |
| 3 | Arg/D-Val | 7.5 | 8.6 | 11.0 | 10.5 |
| 4 | Pro/D-Pro | - | - | - | - |
| 5 | Pro/D-Ser | - | *b.s.* | - | 11.5 |
| 6 | Met/D-Trp | 7.1 | *b.s.* | 10.0 | 12.0 |
| 7 | Trp/D-Met | 7.3 | 8.0 | 8.0 | 4.0 |
| 8 | Ser/D-Pro | 7.7 | - | 4.0 | - |
| 9 | Pro/D-Pro | - | - | - | - |
| 10 | Val/D-Arg | 8.5 | 7.0 | 8.5 | 8.5 |
| 11 | Trp/D-His | 6.8 | *b.s.* | 10.0 | 9.5 |
| 12 | Pro/D-Thr | - | 8.8 | - | 9.0 |
|  |  |  |  |  |  |

*o. ≡ overlapped*

*n.o. ≡ not observed*

*b.s. ≡ broad signal*

**Table 5**. Proportion of each conformational species of each peptide (obtained by integrating the δPro signals of *trans* and *cis* conformers).

| Peptide | Proportion (%) | Xaa Pro conformer |
| --- | --- | --- |
|  |  |  |
| HAI | 23.8 | *cis* |
|  | 76.2 | *trans* |
|  |  |  |
| *retro*-D-HAI | 10.2 | *cis* |
|  | 89.8 | *trans* |
|  |  |  |
| THR | 33.4 | other |
|  | 66.4 | *trans/trans/trans* |
|  |  |  |
| *retro*-D-THR | 7.6 | other |
|  | 92.4 | *trans/trans/trans/trans* |
|  |  |  |

**Computational Analysis**

The essential dynamics method converts atomic fluctuations of C_α_ atoms from the Cartesian space to a set of eigenvectors that describes collective motions that are represented by a list of eigenvectors and their corresponding eigenvalues. To compute the similarity, $\xi_{AB}$, between simulations A and B, Eq. 1[^4^](#_ENREF_4) was used:

$\xi_{AB}=\frac{2\sum_{i=1}^{i=z} \sum_{j=1}^{j=z} \left\{ \left( v_{i}^{A}v_{j}^{B} \right)\frac{exp\left[ -\frac{\left( \Delta x \right)^{2}}{\lambda_{i}^{A}}-\frac{\left( \Delta x \right)^{2}}{\lambda_{j}^{B}} \right]}{\sum_{i=1}^{i=z} exp\left[ -\frac{\left( \Delta x \right)^{2}}{\lambda_{i}^{A}} \right]\sum_{j=1}^{j=z} \left[ -\frac{\left( \Delta x \right)^{2}}{\lambda_{j}^{B}} \right]} \right\}^{2}}{\sum_{i=1}^{i=z} \left\{ \frac{exp\left[ -2\frac{\left( \Delta x \right)^{2}}{\lambda_{i}^{A}} \right]}{\left( \sum_{i=1}^{i=z} exp\left[ -\frac{\left( \Delta x \right)^{2}}{\lambda_{i}^{A}} \right] \right)^{2}} \right\}^{2}+ \sum_{j=1}^{j=z} \left\{ \frac{exp\left[ -2\frac{\left( \Delta x \right)^{2}}{\lambda_{j}^{B}} \right]}{\left( \sum_{j=1}^{j=z} exp\left[ -\frac{\left( \Delta x \right)^{2}}{\lambda_{j}^{B}} \right] \right)^{2}} \right\}^{2}}$ (1)

where $\lambda_{i}$ is an eigenvalue (in Å^2^) associated with eigenvector $\mu_{i}$, whose unitary vector is $v_{i}$. The sum can be extended to all ($z=m$) or a relevant set ($z=n$) of eigenvectors[^4^](#_ENREF_4). The top eigenvectors (*i.e.* those describing the largest collective motions), which explain at least 80% of the accumulated variance, were selected to obtain the similarity index, $\xi_{AB}$.

The similarity obtained for the last 100 ns of each simulation was 74% and 48% for HAI and THR (compared to their *retro*-D-versions), respectively, when only 5 eigenvectors were deployed. As comparative similarity threshold, values around 60-65% are well-accepted when comparing molecular dynamics simulations of large protein systems[^4^](#_ENREF_4). Considering the size of the peptides simulated herein, as well as their internal flexibility and lack of secondary/tertiary structure in solution, the similarity is high –the conformational ensembles of each system are almost half of the time exchangeable each other, thereby supporting the notion that parent and retro-D-peptides adopt similar topological re-arrangements.

**
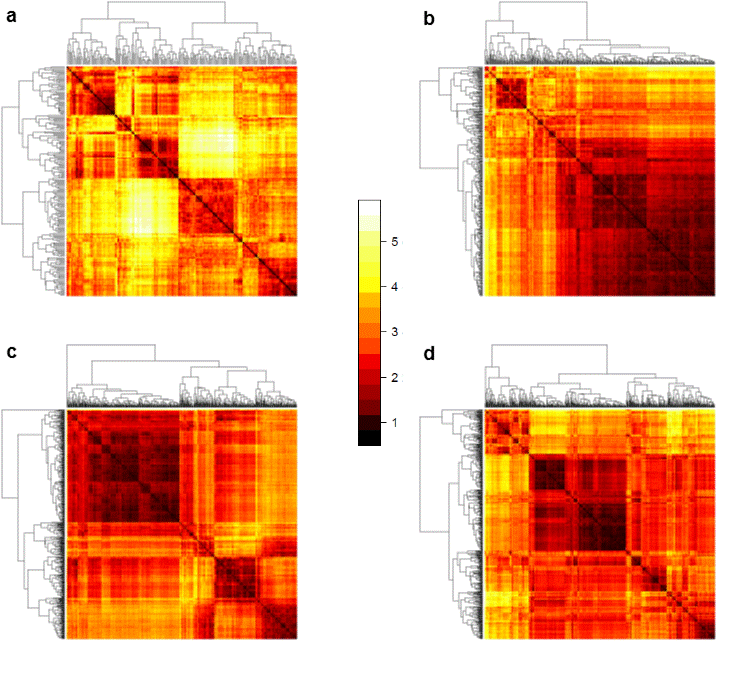
**

**Figure 4.** 2D RMSD plots (mass weighted) for the same 1,000 equally spaced snapshots of each simulation derived from the symmetric RMSD distance matrix computed for (**a**) HAI, (**b**) *retro*-D-HAI, (**c**) THR, and (**d**) *retro*-D-THR.

**Peptide-Protein Conjugate Characterization**

**Figure 5a.** MALDI-TOF spectra of peptide-protein conjugates: (**a**) HAI-BSA, (**b**) THR-BSA (**c**) *retro*-D-HAI-BSA, (**d**) *retro*-D-THR-BSA, (**e**) *retro*-D-HAI-Dpr-BSA, (**f**) *retro*-D-THR-Dpr-BSA, and (**g**) unconjugated BSA.


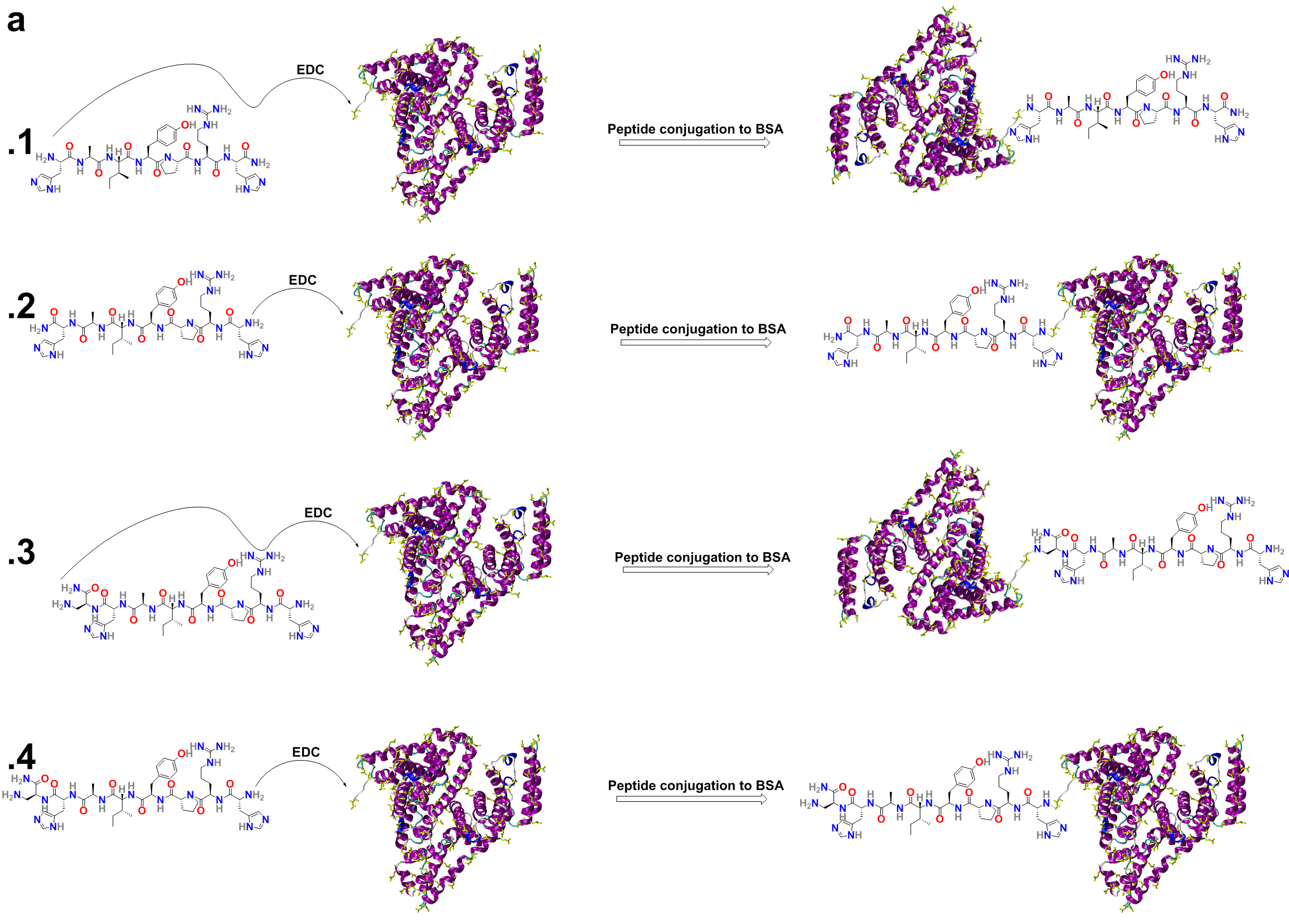


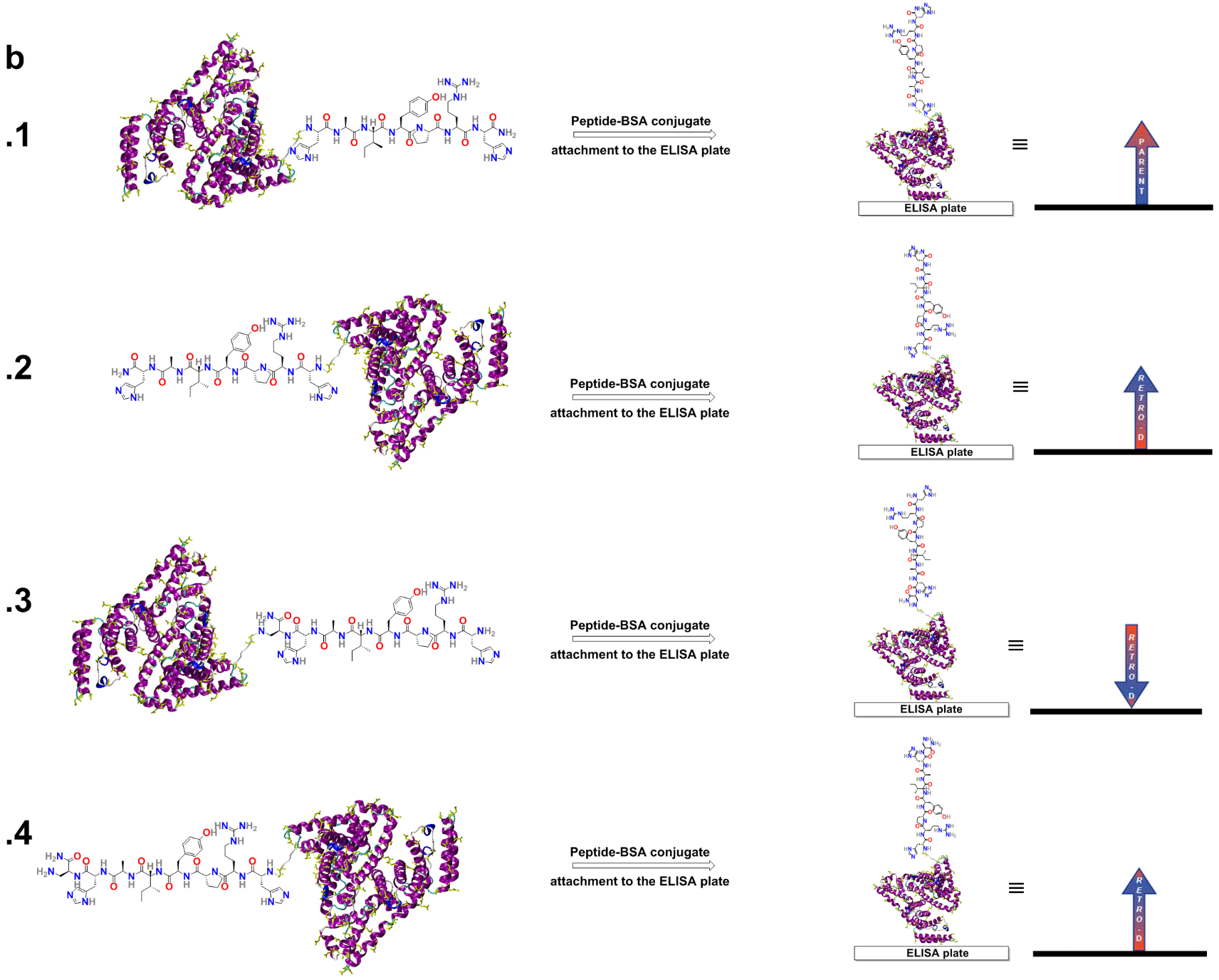


**Figure 5b.** HAI (**.1)**, *retro*-D-HAI (**.2**) and *retro*-D-HAI-Dpr (**.3-4**) conjugation to the ELISA plate: first, (**a**) peptide conjugation to BSA mediated by EDC, and second, (**b**) attachment of the peptide-BSA conjugate to the ELISA plate.

**Mouse Immunization Chronology**

**Scheme 1.** Chronology of mouse immunization experiments.

**Rabbit Immunization Chronology**

**Scheme 2.** Chronology of rabbit immunization experiments.

**Immunological Study in Rabbit**

**
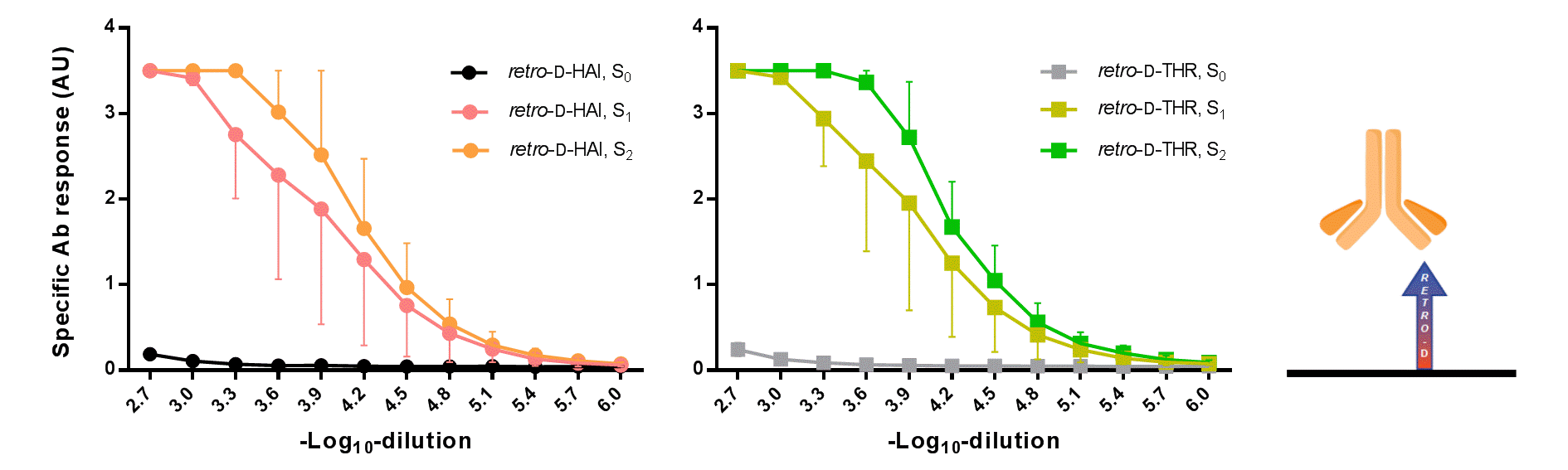
**

**Figure 6**. Titration of humoral response by ELISA in rabbit: serum anti-*retro*-D-peptides conjugated to KLH.

**Rabbit Polyclonal Antibody Purification and Characterization**

**Figure 7**. Characterization of pAbs against *retro*-D-peptides produced in rabbit after affinity purification: (**a**) pAb titration with ELISA and (**b**) SDS-PAGE.

**Other Supplementary Information**

**Figure 8**. Strategy to design unrecognizable therapeutic viruses with modulated tropism: through the attachment of (**a**) a *retro*-D-peptide + linker or of (**b**) a *retro*-D-peptide, directly.

**Abbreviations**

^1^H-NMR proton nuclear magnetic resonance

AAV adeno-associated virus

BBB blood-brain barrier

BSA bovine serum albumin

CD circular dichroism

CFA complete Freund’s adjuvant

Cl-HOBt 6-chloro-1-hydroxybenzotriazole

CNS central nervous system

CPP cell-penetrating peptides

CSD chemical shift deviation

DCC *N*,*N*'-dicyclohexylcarbodiimide

DHAP 2,6,-dihydroxyacetophenone

DIPCDI *N*,*N*'-diisopropylcarbodiimide

DMF dimethylformamide

Dpr diaminopropionic acid

EDC 1-ethyl-3-(3-dimethylaminopropyl)-carbodiimide

ELISA enzyme-linked immunosorbent assay

Fmoc 9-fluorenylmethoxycarbonyl

HAI H_2_N-HAIYPRH-CONH_2_

HP homing peptide

HRMS high-resolution mass spectrometry

IFA incomplete Freund’s adjuvant

IgG Immunoglobulin G

IgM Immunoglobulin M

KLH keyhole limpet hemocyanin

mAbs monoclonal antibodies

MALDI-TOF MS matrix-assisted laser desorption/ionization time-of-flight mass spectrometry

MHC major histocompatibility complex

MTBE methyl *tert*-butyl ether

NHS *N*-hydroxysuccinimide

NMR nuclear magnetic resonance

pAbs polyclonal antibodies

PBS phosphate-buffered saline

PEG polyethylene glycol

RC random coil

REMD replica exchange molecular dynamics

*retro*-D-HAI H_2_N-hrpyiah-CONH_2_

*retro*-D-THR H_2_N-pwvpswmpprht-CONH_2_

*retro*-D-version *retro*-enantio-/ *retro*-inverso-version of a parent L-peptide

RMSD root-mean-square deviation

RP-HPLC reversed-phase high-performance liquid chromatography

SEM standard error of the mean

SPPS solid-phase peptide synthesis

*t*Bu *tert*-butyl

T20 tween 20

THR H_2_N-THRPPMWSPVWP-CONH_2_

TMB 3,3',5,5'-tetramethylbenzidine

t_R_ retention time

**References**

1. Wüthrich, K. NMR of proteins and nucleic acids. (Wiley, 1986).

2. Schubert, M., Labudde, D., Oschkinat, H. & Schmieder, P. A software tool for the prediction of Xaa-Pro peptide bond conformations in proteins based on 13C chemical shift statistics. *J Biomol NMR* **24**, 149-154 (2002).

3. Wishart, D.S., Bigam, C.G., Holm, A., Hodges, R.S. & Sykes, B.D. 1H, 13C and 15N random coil NMR chemical shifts of the common amino acids. I. Investigations of nearest-neighbor effects. *J Biomol NMR* **5**, 67-81 (1995).

4. Pérez, A. et al. Exploring the essential dynamics of B-DNA. *‎J Chem Theory Comput* **1**, 790-800 (2005).
